# Quantitative Analysis of Timed Up and Go Metrics Across Parkinson’s Disease Severity and Their Clinical Correlations

**DOI:** 10.3390/diagnostics16142283

**Published:** 2026-07-21

**Authors:** Danyeong Kim, Minji Son, Jeanhong Jeon, Da-Eun Jeong, Hyun Kyung Yi, Min-Ju Kang

**Affiliations:** 1 Department of Bionano Technology, Gachon University, Seongnam-si 13120, Gyeonggi-do, Republic of Korea; dan627328@gmail.com; 2 Department of Neurology, Veterans Medical Research Institute, Veterans Health Service Medical Center, Gangdong-gu, Seoul 05368, Republic of Korea; doctorjung86@gmail.com; 3 Research Institute, JEIOS Inc., Busan 46903, Republic of Korea; thsepd87@naver.com (M.S.); jeanhongjeon@gmail.com (J.J.); 4 Department of Nuclear Medicine, Veterans Health Service Medical Center, Gangdong-gu, Seoul 05368, Republic of Korea; hkyinm@bohun.or.kr

**Keywords:** Parkinson’s disease, gait, biomarker, Timed Up and Go, turning

## Abstract

**Background:** Parkinson’s disease (PD) diagnosis is often delayed until signature motor symptoms manifest, at which point profound dopaminergic neuron loss has already occurred, necessitating advanced motor diagnostic biomarkers. Quantitative gait analysis is a promising tool, but phase-specific kinematic parameters remain underexplored. This study aims to identify novel, stage-divided Timed Up and Go (TUG) biomarkers not only to differentiate healthy controls (HCs) from patients with PD but also to objectively monitor and track disease progression across advancing severity stages, which are further validated against conventional clinical motor scales. **Methods:** A total of 81 participants (48 PD, 33 HCs) performed a 3 m TUG test using MotionCore (JEIOS Inc., Busan, Republic of Korea). The test was subdivided into three movement phases (Stage 1, sit-to-walk; Stage 2, turning; Stage 3, walk-to-sit). **Results:** PD patients exhibited significantly prolonged durations and altered turning metrics compared to HCs. Turning parameters including turning radius (ETR), area (EMA), and turning stability (FN) demonstrated strong correlations with disease severity and clinical scales. Notably, stage-specific analyses revealed that step counts, time, and speed metrics across Stages 1, 2, and 3 effectively differentiated disease severity, with transitional decelerating and seating metrics in Stage 3 showing the most pronounced clinical correlations. **Discussion:** This study confirms that the TUG test systematically deteriorates with increasing PD severity. The robust correlations with clinical scales (UPDRS, FOG-Q, BBS) validate TUG metrics as objective measures of motor and balance impairments. Utilizing novel, staging-specific indices significantly enhances the TUG test’s clinical utility for supporting diagnosis, accurate staging, and monitoring disease progression. Although the overall group comparisons demonstrated statistical significance, a data overlap remains between mild PD and HCs, underscoring the need for large-scale longitudinal studies to validate these metrics for early detection.

## 1. Introduction

With the rapid aging of the global population, the prevalence of Parkinson’s disease (PD) is increasing significantly, with projections estimating 25 million cases by 2050 [1]. PD is the second most common neurodegenerative disorder after Alzheimer’s disease [2]. The hallmark motor symptoms of PD include resting tremor, rigidity, bradykinesia, and postural instability [3], alongside various non-motor symptoms, such as hyposmia and sleep disorders [4]. Since these symptoms are often difficult for patients to recognize in the early stages, there is a considerable time lag between symptom onset and clinical diagnosis. Importantly, by the time a diagnosis is confirmed, approximately 50–60% of dopaminergic neurons have already been lost [5,6], emphasizing the need for immediate and highly sensitive monitoring of motor symptom progression.

To address this need, gait analysis has recently gained attention as a promising diagnostic and monitoring tool [7,8,9,10]. Gait disturbances are highly prevalent in PD. Notably, gait difficulty is a primary contributor to functional disability and significantly decreases quality of life (QoL) in patients with mild-to-moderate PD [11]. Therefore, comprehensive gait analysis is crucial not only for supporting diagnosis but also for monitoring disease status and distinguishing severity stages [12,13,14]. Specifically, PD patients experience a shuffling gait, characterized by difficulty lifting the feet from the ground, which impairs leg swing and propulsion [15]. Based on these typical features, previous studies have quantified parameters, such as cadence, stride length, and support time, reporting increased cadence and decreased stride length in PD groups [16,17,18]. Furthermore, changes in gait speed and cadence have been correlated with cognitive status, such as Montreal Cognitive Assessment (MoCA) score [19,20], and brain atrophy in multiple regions has been observed in PD patients exhibiting gait deterioration [21].

However, research focused on discovering novel biomarkers beyond conventional gait parameters remains limited. In particular, comparative studies across different stages of PD severity or longitudinal analyses are scarce [22,23,24], and there is a lack of comprehensive investigations comparing healthy controls (HCs) specifically with early-stage PD groups. In our previous study, we confirmed significant differences in gait metrics, including stride length, during the Timed Up and Go (TUG) test between the PD and HC groups [16].

Building upon our prior findings, this study aims to identify novel TUG-based biomarkers and examine the differences between HCs and various PD severity groups by subdividing the TUG test into specific task stages. Additionally, we evaluated the clinical validity of these metrics by assessing their correlations with motor function scales, such as the Unified Parkinson’s Disease Rating Scale (UPDRS).

## 2. Methods

### 2.1. Patient Collection

The PD and HC groups were recruited from the Veterans Health Service Medical Center. PD patients were enrolled if they met the UK Parkinson’s Disease Society Brain Bank diagnostic criteria for PD, which was confirmed by a neurologist after at least six months of follow-up. Inclusion criteria for the PD group included an age range of 55 to 85 years and confirmation of diagnosis via brain MRI and 18F-FP-CIT PET imaging. Clinical assessments included the MDS-UPDRS Part III, Hoehn and Yahr (H&Y) stage, Freezing of Gait Questionnaire (FOG-Q), and Berg Balance Scale (BBS). Cognitive and psychological status were evaluated using the K-MMSE and Geriatric Depression Scale (GDS). HCs were recruited based on the 28-item criteria for normal aging [25]. To ensure the validity of the gait assessment, HCs were required to be free from additional musculoskeletal or severe neurological disorders that could significantly impair independent mobility. All participants provided written informed consent, and the study was approved by the Institutional Review Board of the Veterans Healthcare Medical Center on 19 September 2024 (2024-08-010).

### 2.2. Measurements of Gait

**Figure 1:**
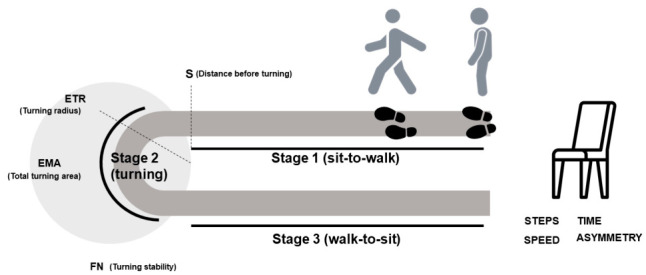
Schematic illustration of the Timed Up and Go (TUG) test. It was subdivided into stages (Stage 1: sit-to-walk, Stage 2: turning, Stage 3: walk-to-sit) and extracted kinematic metrics. Parameters are detailed in Supplementary Table S1.

Gait parameters were measured using MotionCore (JEIOS Inc., Busan, Republic of Korea), a system integrated with a camera-based visualization unit and sensor-equipped sneakers [16,26]. Gait performance was evaluated using the Timed Up and Go (TUG) test. Prior to the formal assessment, each participant’s preferred walking speed was determined using a metronome to ensure a consistent baseline (normal speed). Based on this preferred speed, the TUG test was performed under three different speed conditions: Slow (Normal × 0.8), Normal, and Fast (Normal × 1.2). For each condition, participants started from a seated position on a chair without armrests, stood up upon the start signal, walked 3 m, turned at a designated point, and returned to the seated position (Figure 1). Detailed TUG features were explained in Supplementary Table S1.

### 2.3. Statistical Analysis

Statistical analyses were performed using the Python program (version 3.12.13) utilizing Pandas (version 2.2.2), as well as GraphPad Prism software version 8.0.2 (GraphPad Software Inc., San Diego, CA, USA). Data from the three speed conditions were averaged to ensure data stability and minimize intra-individual variability. Group comparisons between the HC and PD groups were performed using Student’s *t*-test. For multi-group comparisons, a one-way ANOVA followed by Tukey’s post hoc test was conducted. Correlation analysis was conducted using Spearman’s rank correlation coefficient. Statistical significance was defined as *p* < 0.05, and data are presented as mean ± standard deviation (SD).

## 3. Results

### 3.1. Demographic and Clinical Characteristics of Participants

A total of 81 participants, consisting of 48 patients with PD and 33 HCs, were included in this study. The demographic and clinical characteristics of both groups, including statistical comparisons, are comprehensively summarized in Table 1. Within the PD cohort, the average scores for clinical severity were 34 for the UPDRS, 25.1 for the K-MMSE, 7.3 for the FOG-Q, 2.5 for the GDS, and 42 for the BBS.

### 3.2. Significant Changes in TUG Metrics in PD Patients

**Figure 2:**
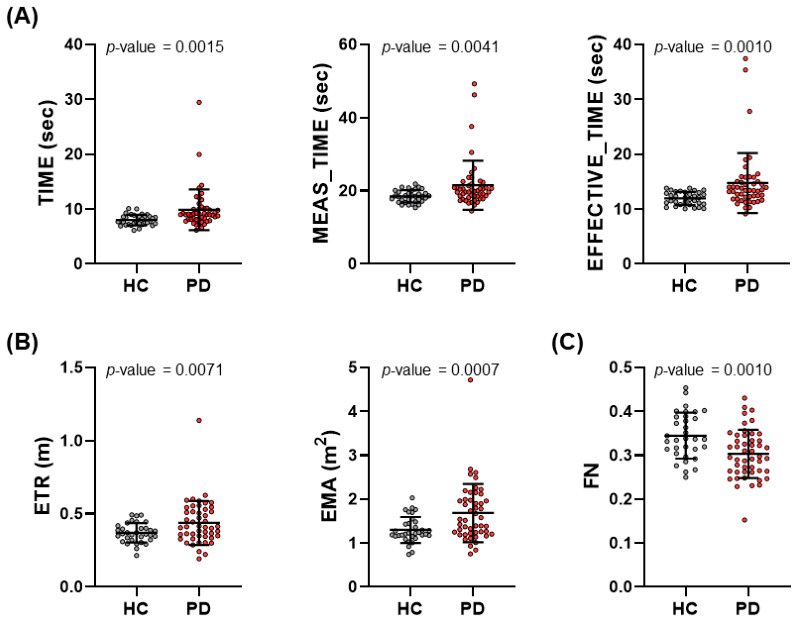
Prolonged duration and altered kinematic metrics in PD patients compared to HCs. (**A**) PD patients exhibited significantly increased total, measured (MEAS), and effective time during the TUG test. (**B**) PD patients showed significant alterations in turning metrics, specifically increased ETR and EMA. (**C**) TUG stability (FN) was significantly decreased in the PD group. *p*-values were calculated using Student’s *t*-test and are presented in Table 2.

Independent *t*-tests were conducted to compare various TUG metrics between the HC and PD groups (Figure 2). The results, detailed in Table 2, indicate significant differences in most TUG features between the two groups. Specifically, the PD patients significantly demonstrated impaired performance, characterized by prolonged times for the test, increased step counts, and reduced gait speed. Crucially, the turning and stability metrics in the TUG test, such as ETR, EMA, and FN, revealed significant differences, underscoring their sensitivity for PD diagnosis. Conversely, some metrics, particularly those related to asymmetry and S, did not show statistical significance between HC and PD groups.

### 3.3. TUG Metric Differences Across H&Y Disease Severity Groups

**Figure 3:**
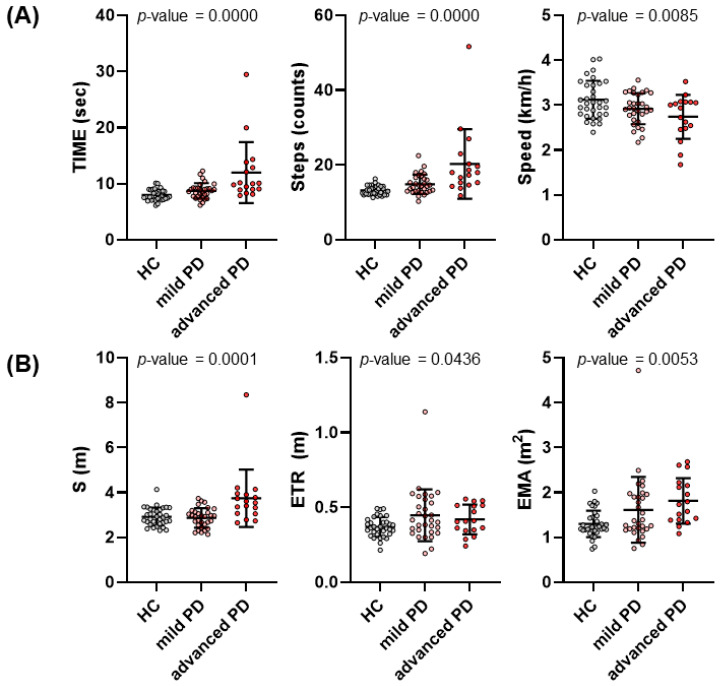
Significant differences in TUG metrics according to PD severity. (**A**) Total duration and step count progressively increased with advancing H&Y stages, whereas gait speed decreased. (**B**) Specific kinematic parameters including turning start distance (S), radius (ETR), and area (EMA) increased depending on disease severity. *p*-values were calculated using one-way ANOVA and are detailed in Table 3.

One-way ANOVA and post hoc Tukey tests were conducted to compare TUG metrics across the HC and mild PD (H&Y 1.0–1.5), and advanced PD (H&Y ≥ 2.0) groups (Figure 3). The results, summarized in Table 3, revealed significant differences for the majority of TUG metrics, showing a clear trend of increasing functional impairment alongside advancing disease severity.

Specifically, time-related metrics (TIME, MEAS_TIME, and EFFECTIVE _TIME) exhibited a highly significant main effect of the group. The analysis indicated that while the mild PD group showed relatively preserved function, the advanced PD group exhibited a sharp increase in these durations. Consistent trends of impairment were observed across a broad spectrum of metrics beyond simple duration. Spatial kinematic control and advanced indices, such as ETR, EMA, and FN, also demonstrated progressive worsening with increasing H&Y stages. These trends were visually confirmed in the box plots in Figure 3, highlighting that the most pronounced deficits occur at H&Y stage 2.0 and above.

### 3.4. Stage-Specific TUG Metric Comparisons Across H&Y Groups

**Figure 4:**
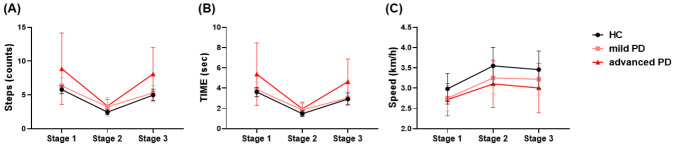
Stage-specific comparisons of step count, duration, and speed across groups. PD patients exhibited a progressive increase in (**A**) step count and (**B**) phase duration across the three TUG stages. (**C**) Gait speed decreased in the PD group, showing a more pronounced decline in the advanced PD group. Increased disease severity contributed to greater task variability across distinct TUG stages. *p*-values were calculated using one-way ANOVA and are detailed in Table 4.

The analysis further investigated how individual TUG stage metrics changed across Stage 1, Stage 2, and Stage 3, stratified by H&Y groups (Table 4). Significant differences across H&Y groups were found for the majority of spatio-temporal metrics, including STAGE1_RealSteps (*p* = 0.0004), STAGE1_TIME (*p* = 0.0005), STAGE1_SPEED (*p* = 0.0142), STAGE2_RealSteps (*p* = 0.0017), STAGE2_TIME (*p* = 0.0029), STAGE2_SPEED (*p* = 0.0036), STAGE3_RealSteps (*p* < 0.0001), STAGE3_TIME (*p* < 0.0001), and STAGE3_SPEED (*p* = 0.0057). As illustrated in the line plots in Figure 4, patients in the advanced PD group consistently demonstrated a general increase in time and steps, alongside a corresponding decrease in speed, across all three individual stages compared to the HC and mild PD groups. Notably, the advanced PD group exhibited higher variability and more pronounced fluctuations across the stages. The increases in duration and step counts were particularly exaggerated during Stage 1 and Stage 3 compared to Stage 2, which is a transitional turning phase. This pattern suggests that motor difficulties in PD are pervasive across all sub-tasks of the TUG test, with the advanced group showing the most pronounced deficits regardless of the specific movement phase.

### 3.5. Correlation Between TUG Metrics and Clinical Variables

**Figure 5:**
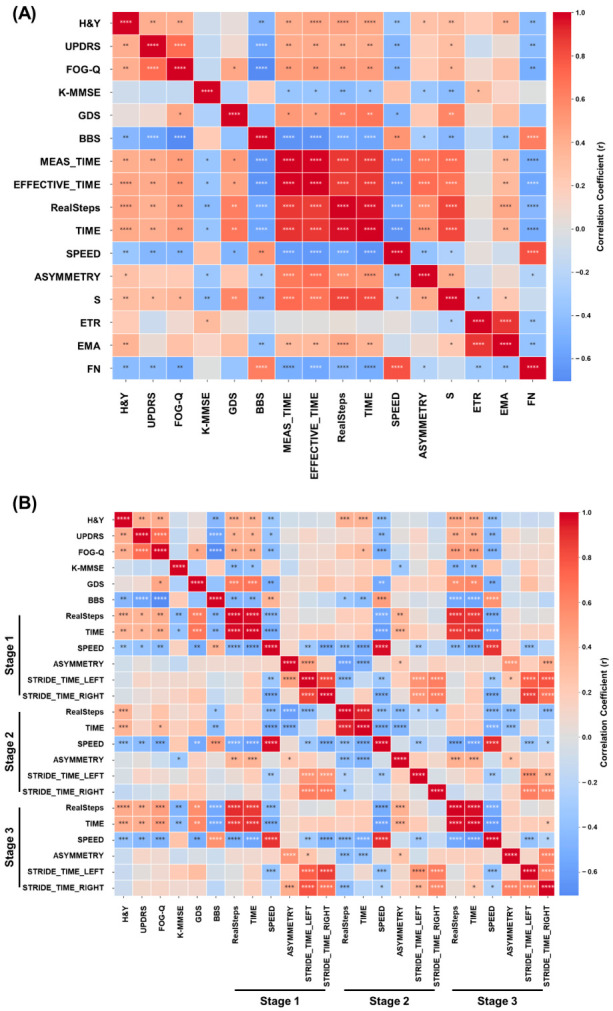
Correlation metrics between clinical variables and TUG metrics. Heatmaps illustrating the correlation coefficients between clinical scales and TUG parameters. (**A**) Correlation with TUG metrics and (**B**) correlation with stage-specific parameters. The color indicates the strength and direction of the correlation, where dark red represents a perfect positive correlation and dark blue represents a strong negative correlation. Statistical significance is indicated by asterisks within the cells. * *p* < 0.05, ** *p* < 0.01, *** *p* < 0.001, **** *p* < 0.0001. Detailed correlation coefficients and *p*-values for all pairs are provided in Supplementary Table S2.

Correlation analyses were performed to examine the relationships between TUG metrics and clinical variables (Figure 5, Supplementary Table S2). Significant positive correlations were observed between TUG temporal metrics and clinical severity indicators, such as MEAS_TIME with H & Y (r = 0.3986, *p* = 0.0002), UPDRS (r = 0.4104, *p* = 0.0038), and FOG-Q (r = 0.4876, *p* = 0.0004). Conversely, these temporal metrics were strongly negatively correlated with the BBS, with TIME exhibiting the most pronounced negative correlation (r = −0.6547, *p* < 0.0001), indicating that superior postural balance is significantly associated with shorter TUG durations. TUG speed metrics, such as speed, exhibited inverse correlation patterns.

As illustrated in the segmented correlation heatmap (Figure 5A), clinical scales focusing on physical symptoms, like UPDRS and FOG-Q, and BBS, consistently demonstrated robust correlations with a wide range of TUG metrics. These findings strongly reinforce the utility of the TUG test and its derived indices as robust, objective tools for assessing motor and balance impairments in PD. Additionally, the cognitive (K-MMSE) and psychological (GDS) scores also exhibited significant correlations with spatio-temporal parameters. This indicated that cognitive–affective factors are also integrated into advanced gait performance.

Furthermore, stage-specific correlation analysis revealed that the clinical relevance of TUG metrics varied depending on the specific movement phase (Figure 5B). Notably, metrics extracted from Stage 3 (walk-to-sit), followed closely by Stage 2 (the turning phase), displayed noticeably stronger correlations with the UPDRS, FOG-Q, and H&Y stage compared with those from Stage 1 (sit-to-walk). This phase-specific dominance highlights that transitional decelerating, turning, and seating motions are particularly reflective of axial rigidity, postural instability, and freezing susceptibility in PD patients.

## 4. Discussion

This study demonstrates that TUG metrics change significantly according to the progression of PD. By identifying novel TUG-derived biomarkers and analyzing stage-specific differences across disease severity, our findings fundamentally broaden and expand the clinical utility of the TUG test beyond its conventional applications. This provides a critical empirical basis for comprehensively evaluating and monitoring motor dysfunction in PD patients through quantitative gait biomarker analysis.

Our results revealed significance in the majority of TUG metrics between HC and PD patients (Figure 2, Table 2), with a distinct trend of increasing functional impairment as H&Y stages advanced (Figure 3, Table 3). These observations align with previous studies establishing the TUG test as a sensitive tool for distinguishing PD patients from HCs [16,27,28]. A primary contribution of this study lies not only in the identification of novel TUG-based indices, such as ETR, EMA, and FN, but also in demonstrating their capacity for objective stage monitoring across advancing disease severity. Unlike traditional measurements that focus solely on total duration, these advanced metrics capture the underlying complexity and efficiency of motion, offering a more subtle view of gait quality.

Furthermore, our stage-specific analysis revealed that motor impairment in PD is not uniform throughout the TUG task but varies according to specific movement phases (Figure 4, Table 4). By subdividing the test into Stage 1 (sit-to-walk), Stage 2 (turning), and Stage 3 (walk-to-sit), we identified that the advanced PD group exhibited the most pronounced stage-to-stage fluctuations, particularly during the transitions across these individual phases. This granular approach confirms that transitional movements, which require complex postural adjustments and axial coordination, are the most vulnerable to PD-related neurodegeneration. The observed trends suggest that stage-specific metrics can identify particular motor deficits that are often masked in overall time-to-completion measurements.

The strong correlations observed between TUG metrics and clinical indicators, including the UPDRS, FOG-Q, and H&Y stages, further reinforce the validity of the TUG test as a robust indicator of motor impairment (Figure 5, Supplementary Table S2). Specifically, the significant correlations with UPDRS and FOG-Q scores suggest that TUG performance deteriorates with the worsening of motor symptoms and freezing of gait. Similarly, postural stability measured by BBS demonstrated extensive correlations across the TUG parameters, aligning consistently with previous studies [16,29]. Time-related metrics exhibited significant positive correlations, confirming that diminished balance prolongs completion times, while gait speed and FN (turning stability) showed negative correlation. Crucially, our stage-specific correlation analysis added more detailed insights to these findings by revealing that the clinical relevance of the TUG test is highly phase-dependent. Several metrics extracted from Stage 3 and Stage 2 displayed noticeably stronger and more extensive correlations with the UPDRS, FOG-Q, H&Y stages, and BBS compared to those from Stage 1. This stage-specific analysis highlights that movement transitions, which require complex body coordination, precise slowing down, and dynamic balance adjustments, are sensitive to the progression of PD. Importantly, the K-MMSE and GDS scores also exhibited meaningful correlations with gait parameters. This association indicates that cognitive–affective factors are also integrated into complex gait control and multi-phase gait control rather than operating independently. These findings are consistent with previous studies demonstrating that cognitive decline is closely linked to gait instability and increased variability in PD [16,30,31]. Collectively, these comprehensive and phase-sensitive associations highlight that the clinical relevance of the TUG test is sensitive to the progression of PD for assessing functional mobility, precise deceleration, and dynamic postural adjustments.

Despite providing valuable insights into TUG metrics in PD, this study has several limitations. First, there were discrepancies in age and sex distribution between the PD and HC groups. While we achieved statistical significance with 81 participants, a larger and more diverse cohort would further enhance the robustness and generalizability of the results. Second, the cross-sectional design limits the ability to track individual disease progression and longitudinal changes in TUG metrics over time. In particular, although we observed statistically significant differences between the HC and PD groups, an overlap in data points remains between HCs and patients with mild PD. Therefore, further studies with a larger and more detailed cohort stratified by precise disease stages are needed. Furthermore, incorporating longitudinal data will be essential to track subtle within-individual biomechanical alterations over time, which could eventually help establish reliable diagnostic cut-off values for early-stage detection and extract integrated diagnostic value.

Finally, although we assessed cognitive function using the K-MMSE, we did not incorporate the MoCA, which may be more sensitive to subtle cognitive deficits in PD. Future research including a broader range of cognitive assessments would further strengthen these findings.

In conclusion, this study demonstrates significant differences in TUG metrics between HC and PD patients. Furthermore, gait performance systemically worsens as disease severity increases, characterized by prolonged duration, increased step count, and reduced speed, as disease severity increases. The strong correlations between TUG metrics and clinical motor scales (UPDRS, FOG-Q, BBS) confirm its utility as an objective tool for assessing motor and balance impairments. Our findings suggest that the TUG test, utilizing novel stage-specific indices, also serves as a valuable and objective instrument in clinical practice for supporting diagnosis, staging disease severity, and monitoring motor progression in PD.

## Figures and Tables

**Table 1:** Demographic and clinical characteristics of study participants.

	HC (*n* = 33)	PD (*n* = 48)	*p*
Age	73 (59–81)	78 (66–85)	0.0001 ^a^
Sex (Male/Female)	23/10	44/4	0.0102 ^b^
Height	165 (146–175)	166 (150–178)	0.6371 ^a^
Weight	66.4 (42.0–90.0)	66.0 (46.0–85.0)	0.9790 ^a^
H&Y Scale (1.0/1.5/>2.0)	-	17/14/17	-
UPDRS	-	34 (4–83)	-
K-MMSE		25.1 (14–30; *n* = 44)	
FOG-Q	-	7.3 (0–22)	-
GDS		2.5 (0–12; *n* = 26)	
BBS	-	42 (16–56)	-

Data are presented as median (range). ^a^ = Mann–Whitney test. ^b^ = Fisher’s test. Abbreviations: HC, healthy control; PD, Parkinson’s disease; H&Y scale, Hoehn and Yahr scale; UPDRS, Unified Parkinson’s Disease Rating Scale; K-MMSE, Korean Mini-Mental State Examination; FOG-Q, Freezing of Gait Questionnaire; GDS, Geriatric Depression Scale; BBS, Berg Balance Scale.

**Table 2:** Comparison of TUG metrics between healthy controls and Parkinson’s disease patients.

TUG Features	HC (Mean ± SD)	PD (Mean ± SD)	*p*-Value
TIME	8.024 ± 0.967	9.893 ± 3.705	**0.0015**
MEAS_TIME	18.486 ± 1.672	21.510 ± 6.692	**0.0041**
EFFECTIVE_TIME	11.943 ± 1.194	14.765 ± 5.444	**0.0010**
RealSteps	13.207 ± 1.216	16.813 ± 6.382	**0.0004**
TIME	8.024 ± 0.967	9.893 ± 3.705	**0.0015**
SPEED	3.121 ± 0.422	2.858 ± 0.406	**0.0067**
ASYMMETRY	1.009 ± 0.005	1.013 ± 0.015	0.0506
S	2.925 ± 0.403	3.181 ± 0.926	0.0949
ETR	0.370 ± 0.066	0.438 ± 0.150	**0.0071**
EMA	1.300 ± 0.294	1.685 ± 0.664	**0.0007**
FN	0.345 ± 0.052	0.303 ± 0.055	**0.0010**

Data are presented as mean ± standard deviation (SD). *p*-values were determined using the independent *t*-test to compare the HC and PD groups. Abbreviation: HC, healthy control; PD, Parkinson’s disease; MEAS_TIME, measured time. Bold values indicate statistically significant differences (*p* < 0.05).

**Table 3:** Comparison of TUG metrics across Hoehn and Yahr (H&Y) disease severity groups.

TUG Feature	HC (Mean ± Std)	Mild PD (Mean ± Std)	Advanced PD (Mean ± Std)	*p*-Value
TIME	8.024 ± 0.967	8.748 ± 1.384	11.982 ± 5.435	**0.0000**
MEAS_TIME	18.486 ± 1.672	19.660 ± 2.385	24.882 ± 10.108	**0.0002**
EFFECTIVE_TIME	11.943 ± 1.194	13.147 ± 1.948	17.716 ± 8.101	**0.0000**
RealSteps	13.207 ± 1.216	14.892 ± 2.595	20.314 ± 9.319	**0.0000**
SPEED	3.121 ± 0.422	2.921 ± 0.345	2.742 ± 0.490	**0.0085**
ASYMMETRY	1.009 ± 0.005	1.010 ± 0.004	1.019 ± 0.024	**0.0066**
S	2.925 ± 0.403	2.872 ± 0.435	3.744 ± 1.280	**0.0001**
ETR	0.370 ± 0.066	0.448 ± 0.173	0.420 ± 0.099	**0.0436**
EMA	1.300 ± 0.294	1.615 ± 0.735	1.813 ± 0.507	**0.0053**
FN	0.345 ± 0.052	0.316 ± 0.050	0.281 ± 0.058	**0.0005**

Data are presented as mean ± standard deviation (SD). *p*-values were determined using one-way ANOVA followed by Tukey’s post hoc test. Abbreviation: HC, healthy control; PD, Parkinson’s disease (mild PD: H&Y stages 1.0–1.5; advanced PD: H&Y stages ≥ 2.0); MEAS_TIME, measured time. Bold values indicate statistically significant differences (*p* < 0.05).

**Table 4:** Comparison of stage-specific TUG metrics across Hoehn and Yahr (H&Y) disease severity groups.

TUG Feature	HC (Mean ± Std)	Mild PD (Mean ± Std)	Advanced PD (Mean ± Std)	*p*-Value
STAGE1_RealSteps	5.778 ± 0.578	6.349 ± 1.180	8.873 ± 5.278	**0.0004**
STAGE1_TIME	3.621 ± 0.441	3.862 ± 0.743	5.383 ± 3.077	**0.0005**
STAGE1_SPEED	2.983 ± 0.376	2.754 ± 0.317	2.717 ± 0.401	**0.0142**
STAGE1_ASYMMETRY	1.025 ± 0.014	1.011 ± 0.094	1.023 ± 0.054	0.6536
STAGE1_STRIDE_TIME_LEFT	1.161 ± 0.111	1.123 ± 0.162	1.155 ± 0.114	0.4986
STAGE1_STRIDE_TIME_RIGHT	1.153 ± 0.107	1.129 ± 0.109	1.141 ± 0.094	0.6534
STAGE2_RealSteps	2.455 ± 0.378	3.231 ± 1.349	3.353 ± 0.998	**0.0017**
STAGE2_TIME	1.479 ± 0.286	1.845 ± 0.631	1.966 ± 0.643	**0.0029**
STAGE2_SPEED	3.550 ± 0.458	3.254 ± 0.395	3.106 ± 0.582	**0.0036**
STAGE2_ASYMMETRY	1.461 ± 0.045	1.431 ± 0.075	1.466 ± 0.155	0.2857
STAGE2_STRIDE_TIME_LEFT	1.452 ± 0.163	1.425 ± 0.242	1.426 ± 0.246	0.8575
STAGE2_STRIDE_TIME_RIGHT	1.507 ± 0.247	1.389 ± 0.240	1.442 ± 0.184	0.1330
STAGE3_RealSteps	4.975 ± 0.878	5.312 ± 1.082	8.088 ± 3.928	**0.0000**
STAGE3_TIME	2.925 ± 0.589	3.040 ± 0.674	4.634 ± 2.239	**0.0000**
STAGE3_SPEED	3.458 ± 0.457	3.220 ± 0.389	3.004 ± 0.612	**0.0057**
STAGE3_ASYMMETRY	1.025 ± 0.014	0.963 ± 0.167	1.013 ± 0.046	0.0615
STAGE3_STRIDE_TIME_LEFT	1.150 ± 0.103	1.118 ± 0.113	1.124 ± 0.080	0.4317
STAGE3_STRIDE_TIME_RIGHT	1.151 ± 0.102	1.065 ± 0.201	1.130 ± 0.092	0.0655

Data are presented as mean ± standard deviation (SD). *p*-values were determined using one-way ANOVA followed by Tukey’s post hoc test. Abbreviation: HC, healthy control; PD, Parkinson’s disease (mild PD: H&Y stages 1.0–1.5; advanced PD: H&Y stages ≥ 2.0); Stage 1, sit-to-walk phase; Stage 2, turning phase; Stage 3, walk-to-sit phase. Bold values indicate statistically significant differences (*p* < 0.05).

## Data Availability

The datasets used and analyzed during the current study are available from the corresponding author upon reasonable request.

## Supplementary Materials

The following supporting information can be downloaded at: https://www.mdpi.com/article/10.3390/diagnostics16142283/s1, Table S1: Description of the extracted TUG parameters; Table S2: Pearson correlation coefficients and *p*-values among clinical scales and TUG metrics.
