# Supplementary material for: Quantitative Analysis of Timed Up and Go Metrics Across Parkinson’s Disease Severity and Their Clinical Correlations
Source: Diagnostics (Basel). 2026 Jul 21;16(14):2283. doi: 10.3390/diagnostics16142283 (PMC13408167; doi:10.3390/diagnostics16142283)
Supplement: Supplementary file 1 [file diagnostics-16-02283-s001.zip › diagnostics-4374363-supplementary.pdf]

Quantitative Analysis of Timed Up and Go Metrics Across Parkinson's Disease Severity and Their Clinical Correlations

Danyeong Kim[1,2], Minji Son[3], Jeanhong Jeon[3], Da-Eun Jeong[2], Hyun Kyung Yi[4], Min-Ju Kang[2*]

[1] Department of Bionano Technology, Gachon University, Seongnam-si 13120, Gyeonggi-do, Republic of Korea; dan627328@gmail.com

[2] Department of Neurology, Veterans Medical Research Institute, Veterans Health Service Medical Center, Gangdong-gu, Seoul 05368, Republic of Korea; doctorjung86@gmail.com

[3] Research Institute, JEIOS Inc., Busan 46903, Republic of Korea; thsepd87@naver.com (M.S.); jeanhongjeon@gmail.com (J.J.)

[4] Department of Nuclear Medicine, Veterans Health Service Medical Center, Gangdong-gu, Seoul 05368, Republic of Korea; hkyinm@bohun.or.kr

Correspondence: minju.kang@bohun.or.kr; Tel.: +82-2-2225-4547

Supplementary Table S1. Description of the extracted TUG parameters.

Parameter	Description
TIME	Total duration from the initiation of the sit-to-walk phase to the completion of the walk-to-sit phase.
MEAS_TIME	Total measured duration of the TUG trial protocol.
EFFECTIVE_TIME	Total net effective gait time excluding postural preparation or recovery delays.
RealSteps	Total number of steps taken from the movement the participant stands up until they sit back down on the chair.
SPEED	Average gait speed throughout the entire TUG test (km/h).
ASYMMETRY	An index quantifying the spatio-temporal gait asymmetry between left and right steps.
STAGE1_RealSteps	Number of steps taken during the initial sit-to-walk and straight gait phase.
STAGE1_TIME	Total duration of the first phase (sit-to-walk).
STAGE1_SPEED	Average gait speed during the first phase.
STAGE1_ASYMMETRY	Gait asymmetry index calculated specifically for the first phase.
STAGE1_STRIDE_TIME_LEFT	Average stride time of the left lower limb during the first phase.
STAGE1_STRIDE_TIME_RIGHT	Average stride time of the right lower limb during the first phase.
STAGE2_RealSteps	Number of steps taken during the turning (directional transition) phase.
STAGE2_TIME	Total duration of the second phase (turning).
STAGE2_SPEED	Average gait speed during the turning phase.
STAGE2_ASYMMETRY	Gait asymmetry index calculated specifically for the turning phase.
STAGE2_STRIDE_TIME_LEFT	Average stride time of the left lower limb during the turning phase.
STAGE2_STRIDE_TIME_RIGHT	Average stride time of the right lower limb during the turning phase.
STAGE3_RealSteps	Number of steps taken during the return straight gait and walk-to-sit phase.
STAGE3_TIME	Total duration of the third phase (walk-to-sit).
STAGE3_SPEED	Average gait speed during the third phase.
STAGE3_ASYMMETRY	Gait asymmetry index calculated specifically for the third phase.
STAGE3_STRIDE_TIME_LEFT	Average stride time of the left lower limb during the third phase.
STAGE3_STRIDE_TIME_RIGHT	Average stride time of the right lower limb during the third phase.
S	The linear straight-line walking distance covered from standing up immediately before initiating the turn toward the target.
ETR	The kinematic turning radius measured during the directional change at the target.
EMA	The two-dimensional spatial area occupied by the participant's entire gait trajectory during the turning maneuver.
FN(TUG)	Turning stability index, where higher scores reflect smoother, more coordinated, and robust multi-directional foot-strike patterns.

Supplementary Table S2. Pearson correlation coefficients and *p*-values among clinical scales and TUG metrics. Bold values indicate statistically significant differences ($p < 0.05$).

Variable 1	Variable 2	Pearson r	*p*-value
H & Y	UPDRS	0.389	**0.0063**
H & Y	FOG-Q	0.392	**0.0059**
H & Y	K-MMSE	-0.0888	0.5664
H & Y	GDS	0.0399	0.8465
H & Y	BBS	-0.4498	**0.0013**
H & Y	MEAS_TIME	0.3986	**0.0002**
H & Y	EFFECTIVE_TIME	0.4382	**0.0000**
H & Y	RealSteps	0.4513	**0.0000**
H & Y	TIME	0.4276	**0.0001**
H & Y	SPEED	-0.3505	**0.0013**
H & Y	ASYMMETRY	0.2778	0.0120
H & Y	S	0.3133	**0.0044**
H & Y	ETR	0.2177	0.0509
H & Y	EMA	0.3644	**0.0008**
H & Y	FN	-0.4177	**0.0001**
H & Y	STAGE1_RealSteps	0.3659	**0.0008**
H & Y	STAGE1_TIME	0.3513	**0.0013**
H & Y	STAGE1_SPEED	-0.3187	**0.0037**
H & Y	STAGE1_ASYMMETRY	-0.0839	0.4566
H & Y	STAGE1_STRIDE_TIME_LEFT	-0.0546	0.6281
H & Y	STAGE1_STRIDE_TIME_RIGHT	-0.0443	0.6948
H & Y	STAGE2_RealSteps	0.3755	**0.0006**
H & Y	STAGE2_TIME	0.3726	**0.0006**
H & Y	STAGE2_SPEED	-0.3768	**0.0005**
H & Y	STAGE2_ASYMMETRY	-0.0428	0.7041
H & Y	STAGE2_STRIDE_TIME_LEFT	-0.0386	0.7324
H & Y	STAGE2_STRIDE_TIME_RIGHT	-0.1594	0.1552
H & Y	STAGE3_RealSteps	0.4208	**0.0001**
H & Y	STAGE3_TIME	0.3945	**0.0003**
H & Y	STAGE3_SPEED	-0.3651	**0.0008**
H & Y	STAGE3_ASYMMETRY	-0.1196	0.2877
H & Y	STAGE3_STRIDE_TIME_LEFT	-0.1308	0.2446
H & Y	STAGE3_STRIDE_TIME_RIGHT	-0.1108	0.3246
UPDRS	FOG-Q	0.6983	**0.0000**
UPDRS	K-MMSE	-0.1575	0.3073
UPDRS	GDS	0.0859	0.6766

UPDRS	BBS	-0.5744	**0.0000**
UPDRS	MEAS_TIME	0.4104	**0.0038**
UPDRS	EFFECTIVE_TIME	0.4176	**0.0031**
UPDRS	RealSteps	0.3839	**0.0071**
UPDRS	TIME	0.3892	**0.0063**
UPDRS	SPEED	-0.4546	**0.0012**
UPDRS	ASYMMETRY	0.194	0.1865
UPDRS	S	0.3125	**0.0306**
UPDRS	ETR	-0.1051	0.4773
UPDRS	EMA	0.0572	0.6996
UPDRS	FN	-0.4281	**0.0024**
UPDRS	STAGE1_RealSteps	0.3632	**0.0112**
UPDRS	STAGE1_TIME	0.355	**0.0133**
UPDRS	STAGE1_SPEED	-0.3677	**0.0101**
UPDRS	STAGE1_ASYMMETRY	0.0225	0.8791
UPDRS	STAGE1_STRIDE_TIME_LEFT	0.1272	0.3891
UPDRS	STAGE1_STRIDE_TIME_RIGHT	0.0367	0.8043
UPDRS	STAGE2_RealSteps	0.0864	0.5594
UPDRS	STAGE2_TIME	0.1443	0.3277
UPDRS	STAGE2_SPEED	-0.4233	**0.0027**
UPDRS	STAGE2_ASYMMETRY	0.0205	0.8898
UPDRS	STAGE2_STRIDE_TIME_LEFT	0.1314	0.3732
UPDRS	STAGE2_STRIDE_TIME_RIGHT	-0.087	0.5567
UPDRS	STAGE3_RealSteps	0.3916	**0.0059**
UPDRS	STAGE3_TIME	0.3916	**0.0059**
UPDRS	STAGE3_SPEED	-0.4386	**0.0018**
UPDRS	STAGE3_ASYMMETRY	0.1163	0.4310
UPDRS	STAGE3_STRIDE_TIME_LEFT	0.0215	0.8848
UPDRS	STAGE3_STRIDE_TIME_RIGHT	0.1052	0.4768
FOG-Q	K-MMSE	-0.1524	0.3235
FOG-Q	GDS	0.4401	**0.0245**
FOG-Q	BBS	-0.7034	**0.0000**
FOG-Q	MEAS_TIME	0.4876	**0.0004**
FOG-Q	EFFECTIVE_TIME	0.4917	**0.0004**
FOG-Q	RealSteps	0.476	**0.0006**
FOG-Q	TIME	0.4787	**0.0006**
FOG-Q	SPEED	-0.4618	**0.0010**
FOG-Q	ASYMMETRY	0.2021	0.1684
FOG-Q	S	0.3536	**0.0137**
FOG-Q	ETR	0.0866	0.5581

FOG-Q	EMA	0.2696	0.0638
FOG-Q	FN	-0.5041	**0.0003**
FOG-Q	STAGE1_RealSteps	0.4045	**0.0044**
FOG-Q	STAGE1_TIME	0.3982	**0.0051**
FOG-Q	STAGE1_SPEED	-0.4266	**0.0025**
FOG-Q	STAGE1_ASYMMETRY	-0.0717	0.6281
FOG-Q	STAGE1_STRIDE_TIME_LEFT	0.0848	0.5667
FOG-Q	STAGE1_STRIDE_TIME_RIGHT	0.084	0.5703
FOG-Q	STAGE2_RealSteps	0.256	0.0791
FOG-Q	STAGE2_TIME	0.3234	**0.0250**
FOG-Q	STAGE2_SPEED	-0.4949	**0.0003**
FOG-Q	STAGE2_ASYMMETRY	-0.0971	0.5114
FOG-Q	STAGE2_STRIDE_TIME_LEFT	0.0756	0.6096
FOG-Q	STAGE2_STRIDE_TIME_RIGHT	-0.048	0.7459
FOG-Q	STAGE3_RealSteps	0.4767	**0.0006**
FOG-Q	STAGE3_TIME	0.473	**0.0007**
FOG-Q	STAGE3_SPEED	-0.4733	**0.0007**
FOG-Q	STAGE3_ASYMMETRY	0.0852	0.5648
FOG-Q	STAGE3_STRIDE_TIME_LEFT	0.0852	0.5648
FOG-Q	STAGE3_STRIDE_TIME_RIGHT	0.118	0.4246
K-MMSE	GDS	-0.2566	0.2057
K-MMSE	BBS	0.2057	0.1804
K-MMSE	MEAS_TIME	-0.3574	**0.0172**
K-MMSE	EFFECTIVE_TIME	-0.3547	**0.0182**
K-MMSE	RealSteps	-0.4188	**0.0047**
K-MMSE	TIME	-0.3576	**0.0172**
K-MMSE	SPEED	0.2709	0.0753
K-MMSE	ASYMMETRY	-0.3293	**0.0291**
K-MMSE	S	-0.4182	**0.0047**
K-MMSE	ETR	0.3186	**0.0351**
K-MMSE	EMA	0.1349	0.3826
K-MMSE	FN	-0.0025	0.9873
K-MMSE	STAGE1_RealSteps	-0.4241	**0.0041**
K-MMSE	STAGE1_TIME	-0.3787	**0.0113**
K-MMSE	STAGE1_SPEED	0.1506	0.3290
K-MMSE	STAGE1_ASYMMETRY	-0.156	0.3118
K-MMSE	STAGE1_STRIDE_TIME_LEFT	0.0903	0.5599
K-MMSE	STAGE1_STRIDE_TIME_RIGHT	0.1378	0.3724
K-MMSE	STAGE2_RealSteps	0.0803	0.6044
K-MMSE	STAGE2_TIME	0.1736	0.2597

K-MMSE	STAGE2_SPEED	0.2257	0.1406
K-MMSE	STAGE2_ASYMMETRY	-0.3254	**0.0311**
K-MMSE	STAGE2_STRIDE_TIME_LEFT	0.0054	0.9725
K-MMSE	STAGE2_STRIDE_TIME_RIGHT	0.1893	0.2185
K-MMSE	STAGE3_RealSteps	-0.4651	**0.0015**
K-MMSE	STAGE3_TIME	-0.4104	**0.0057**
K-MMSE	STAGE3_SPEED	0.2559	0.0936
K-MMSE	STAGE3_ASYMMETRY	-0.1004	0.5167
K-MMSE	STAGE3_STRIDE_TIME_LEFT	0.2038	0.1845
K-MMSE	STAGE3_STRIDE_TIME_RIGHT	0.0753	0.6272
GDS	BBS	-0.373	0.0605
GDS	MEAS_TIME	0.493	**0.0105**
GDS	EFFECTIVE_TIME	0.4606	**0.0179**
GDS	RealSteps	0.6214	**0.0007**
GDS	TIME	0.6649	**0.0002**
GDS	SPEED	-0.4704	**0.0153**
GDS	ASYMMETRY	0.2292	0.2600
GDS	S	0.5886	**0.0016**
GDS	ETR	0.0605	0.7689
GDS	EMA	0.3116	0.1212
GDS	FN	-0.3585	**0.0721**
GDS	STAGE1_RealSteps	0.6426	**0.0004**
GDS	STAGE1_TIME	0.6668	**0.0002**
GDS	STAGE1_SPEED	-0.5072	**0.0082**
GDS	STAGE1_ASYMMETRY	0.067	0.7451
GDS	STAGE1_STRIDE_TIME_LEFT	0.1072	0.6020
GDS	STAGE1_STRIDE_TIME_RIGHT	0.251	0.2161
GDS	STAGE2_RealSteps	0.159	0.4377
GDS	STAGE2_TIME	0.263	0.1942
GDS	STAGE2_SPEED	-0.5471	**0.0038**
GDS	STAGE2_ASYMMETRY	0.1612	0.4314
GDS	STAGE2_STRIDE_TIME_LEFT	0.1722	0.4002
GDS	STAGE2_STRIDE_TIME_RIGHT	0.1941	0.3421
GDS	STAGE3_RealSteps	0.5596	**0.0030**
GDS	STAGE3_TIME	0.5892	**0.0015**
GDS	STAGE3_SPEED	-0.5039	**0.0087**
GDS	STAGE3_ASYMMETRY	-0.0367	0.8586
GDS	STAGE3_STRIDE_TIME_LEFT	0.3041	0.1310
GDS	STAGE3_STRIDE_TIME_RIGHT	0.1002	0.6263
BBS	MEAS_TIME	-0.6547	**0.0000**

BBS	EFFECTIVE_TIME	-0.6586	**0.0000**
BBS	RealSteps	-0.5488	**0.0001**
BBS	TIME	-0.5601	**0.0000**
BBS	SPEED	0.5226	**0.0001**
BBS	ASYMMETRY	-0.2914	**0.0445**
BBS	S	-0.4463	**0.0015**
BBS	ETR	-0.1346	0.3616
BBS	EMA	-0.375	**0.0086**
BBS	FN	0.6255	**0.0000**
BBS	STAGE1_RealSteps	-0.4536	**0.0012**
BBS	STAGE1_TIME	-0.4504	**0.0013**
BBS	STAGE1_SPEED	0.3734	**0.0090**
BBS	STAGE1_ASYMMETRY	0.0882	0.5513
BBS	STAGE1_STRIDE_TIME_LEFT	-0.0816	0.5815
BBS	STAGE1_STRIDE_TIME_RIGHT	-0.1318	0.3721
BBS	STAGE2_RealSteps	-0.3304	**0.0218**
BBS	STAGE2_TIME	-0.4149	**0.0034**
BBS	STAGE2_SPEED	0.4796	**0.0006**
BBS	STAGE2_ASYMMETRY	0.0257	0.8625
BBS	STAGE2_STRIDE_TIME_LEFT	-0.0675	0.6486
BBS	STAGE2_STRIDE_TIME_RIGHT	-0.024	0.8712
BBS	STAGE3_RealSteps	-0.5497	**0.0001**
BBS	STAGE3_TIME	-0.5588	**0.0000**
BBS	STAGE3_SPEED	0.5415	**0.0001**
BBS	STAGE3_ASYMMETRY	-0.0924	0.5324
BBS	STAGE3_STRIDE_TIME_LEFT	-0.0533	0.7191
BBS	STAGE3_STRIDE_TIME_RIGHT	-0.189	0.1983
MEAS_TIME	EFFECTIVE_TIME	0.9834	**0.0000**
MEAS_TIME	RealSteps	0.8762	**0.0000**
MEAS_TIME	TIME	0.8918	**0.0000**
MEAS_TIME	SPEED	-0.6053	**0.0000**
MEAS_TIME	ASYMMETRY	0.6502	**0.0000**
MEAS_TIME	S	0.7132	**0.0000**
MEAS_TIME	ETR	-0.0076	0.9464
MEAS_TIME	EMA	0.3284	**0.0028**
MEAS_TIME	FN	-0.5043	**0.0000**
EFFECTIVE_TIME	RealSteps	0.8634	**0.0000**
EFFECTIVE_TIME	TIME	0.8746	**0.0000**
EFFECTIVE_TIME	SPEED	-0.6347	**0.0000**
EFFECTIVE_TIME	ASYMMETRY	0.6874	**0.0000**

EFFECTIVE_TIME	S	0.6676	**0.0000**
EFFECTIVE_TIME	ETR	0.0139	0.9017
EFFECTIVE_TIME	EMA	0.3292	**0.0027**
EFFECTIVE_TIME	FN	-0.5509	**0.0000**
RealSteps	TIME	0.9603	**0.0000**
RealSteps	SPEED	-0.5643	**0.0000**
RealSteps	ASYMMETRY	0.5545	**0.0000**
RealSteps	S	0.8362	**0.0000**
RealSteps	ETR	0.0429	0.7040
RealSteps	EMA	0.4295	**0.0001**
RealSteps	FN	-0.446	**0.0000**
TIME	SPEED	-0.6642	**0.0000**
TIME	ASYMMETRY	0.5088	**0.0000**
TIME	S	0.8294	**0.0000**
TIME	ETR	-0.0204	0.8568
TIME	EMA	0.3639	**0.0008**
TIME	FN	-0.5153	**0.0000**
SPEED	ASYMMETRY	-0.372	**0.0006**
SPEED	S	-0.2412	0.0301
SPEED	ETR	0.0233	0.8365
SPEED	EMA	-0.0911	0.4187
SPEED	FN	0.7946	**0.0000**
ASYMMETRY	S	0.3964	**0.0002**
ASYMMETRY	ETR	-0.101	0.3696
ASYMMETRY	EMA	0.0682	0.5452
ASYMMETRY	FN	-0.22	**0.0485**
S	ETR	-0.2397	**0.0312**
S	EMA	0.2284	**0.0403**
S	FN	-0.1187	0.2911
ETR	EMA	0.8834	**0.0000**
ETR	FN	-0.321	**0.0035**
EMA	FN	-0.3708	**0.0007**
STAGE1_RealSteps	STAGE1_TIME	0.9731	**0.0000**
STAGE1_RealSteps	STAGE1_SPEED	-0.4391	**0.0000**
STAGE1_RealSteps	STAGE1_ASYMMETRY	0.0828	0.4624
STAGE1_RealSteps	STAGE1_STRIDE_TIME_LEFT	-0.0057	0.9594
STAGE1_RealSteps	STAGE1_STRIDE_TIME_RIGHT	0.0054	0.9620
STAGE1_RealSteps	STAGE2_RealSteps	0.1789	0.1101
STAGE1_RealSteps	STAGE2_TIME	0.1877	**0.0934**
STAGE1_RealSteps	STAGE2_SPEED	-0.5375	**0.0000**

STAGE1_RealSteps	STAGE2_ASYMMETRY	0.3519	**0.0013**
STAGE1_RealSteps	STAGE2_STRIDE_TIME_LEFT	0.0599	0.5950
STAGE1_RealSteps	STAGE2_STRIDE_TIME_RIGHT	-0.0526	0.6408
STAGE1_RealSteps	STAGE3_RealSteps	0.9112	**0.0000**
STAGE1_RealSteps	STAGE3_TIME	0.8804	**0.0000**
STAGE1_RealSteps	STAGE3_SPEED	-0.4928	**0.0000**
STAGE1_RealSteps	STAGE3_ASYMMETRY	0.0799	0.4782
STAGE1_RealSteps	STAGE3_STRIDE_TIME_LEFT	-0.0149	0.8949
STAGE1_RealSteps	STAGE3_STRIDE_TIME_RIGHT	0.0204	0.8567
STAGE1_TIME	STAGE1_SPEED	-0.521	**0.0000**
STAGE1_TIME	STAGE1_ASYMMETRY	0.1014	0.3676
STAGE1_TIME	STAGE1_STRIDE_TIME_LEFT	0.1922	**0.0856**
STAGE1_TIME	STAGE1_STRIDE_TIME_RIGHT	0.2058	**0.0653**
STAGE1_TIME	STAGE2_RealSteps	0.1318	0.2410
STAGE1_TIME	STAGE2_TIME	0.2019	0.0706
STAGE1_TIME	STAGE2_SPEED	-0.6097	**0.0000**
STAGE1_TIME	STAGE2_ASYMMETRY	0.3593	**0.0010**
STAGE1_TIME	STAGE2_STRIDE_TIME_LEFT	0.19	0.0893
STAGE1_TIME	STAGE2_STRIDE_TIME_RIGHT	0.0815	0.4696
STAGE1_TIME	STAGE3_RealSteps	0.8786	**0.0000**
STAGE1_TIME	STAGE3_TIME	0.8979	**0.0000**
STAGE1_TIME	STAGE3_SPEED	-0.5633	**0.0000**
STAGE1_TIME	STAGE3_ASYMMETRY	0.0837	0.4577
STAGE1_TIME	STAGE3_STRIDE_TIME_LEFT	0.1511	0.1781
STAGE1_TIME	STAGE3_STRIDE_TIME_RIGHT	0.1714	0.1260
STAGE1_SPEED	STAGE1_ASYMMETRY	0.1206	0.2836
STAGE1_SPEED	STAGE1_STRIDE_TIME_LEFT	-0.3276	**0.0028**
STAGE1_SPEED	STAGE1_STRIDE_TIME_RIGHT	-0.5112	**0.0000**
STAGE1_SPEED	STAGE2_RealSteps	-0.412	**0.0001**
STAGE1_SPEED	STAGE2_TIME	-0.5264	**0.0000**
STAGE1_SPEED	STAGE2_SPEED	0.9692	**0.0000**
STAGE1_SPEED	STAGE2_ASYMMETRY	-0.0149	0.8952
STAGE1_SPEED	STAGE2_STRIDE_TIME_LEFT	-0.2903	**0.0086**
STAGE1_SPEED	STAGE2_STRIDE_TIME_RIGHT	-0.1589	0.1566
STAGE1_SPEED	STAGE3_RealSteps	-0.417	**0.0001**
STAGE1_SPEED	STAGE3_TIME	-0.4971	**0.0000**
STAGE1_SPEED	STAGE3_SPEED	0.891	**0.0000**
STAGE1_SPEED	STAGE3_ASYMMETRY	0.1325	0.2384
STAGE1_SPEED	STAGE3_STRIDE_TIME_LEFT	-0.3654	**0.0008**
STAGE1_SPEED	STAGE3_STRIDE_TIME_RIGHT	-0.1779	0.1120

STAGE1_ASYMMETRY	STAGE1_STRIDE_TIME_LEFT	0.4578	**0.0000**
STAGE1_ASYMMETRY	STAGE1_STRIDE_TIME_RIGHT	0.1034	0.3582
STAGE1_ASYMMETRY	STAGE2_RealSteps	-0.5914	**0.0000**
STAGE1_ASYMMETRY	STAGE2_TIME	-0.4795	**0.0000**
STAGE1_ASYMMETRY	STAGE2_SPEED	0.0637	0.5718
STAGE1_ASYMMETRY	STAGE2_ASYMMETRY	0.2347	**0.0350**
STAGE1_ASYMMETRY	STAGE2_STRIDE_TIME_LEFT	0.0366	0.7455
STAGE1_ASYMMETRY	STAGE2_STRIDE_TIME_RIGHT	0.1742	0.1198
STAGE1_ASYMMETRY	STAGE3_RealSteps	0.0844	0.4537
STAGE1_ASYMMETRY	STAGE3_TIME	0.1079	0.3374
STAGE1_ASYMMETRY	STAGE3_SPEED	0.0947	0.4004
STAGE1_ASYMMETRY	STAGE3_ASYMMETRY	0.5308	**0.0000**
STAGE1_ASYMMETRY	STAGE3_STRIDE_TIME_LEFT	0.1801	0.1077
STAGE1_ASYMMETRY	STAGE3_STRIDE_TIME_RIGHT	0.4038	**0.0002**
STAGE1_STRIDE_TIME_LEFT	STAGE1_STRIDE_TIME_RIGHT	0.8443	**0.0000**
STAGE1_STRIDE_TIME_LEFT	STAGE2_RealSteps	-0.4281	**0.0001**
STAGE1_STRIDE_TIME_LEFT	STAGE2_TIME	-0.1423	0.2052
STAGE1_STRIDE_TIME_LEFT	STAGE2_SPEED	-0.3352	**0.0022**
STAGE1_STRIDE_TIME_LEFT	STAGE2_ASYMMETRY	0.1776	0.1127
STAGE1_STRIDE_TIME_LEFT	STAGE2_STRIDE_TIME_LEFT	0.5327	**0.0000**
STAGE1_STRIDE_TIME_LEFT	STAGE2_STRIDE_TIME_RIGHT	0.623	**0.0000**
STAGE1_STRIDE_TIME_LEFT	STAGE3_RealSteps	-0.0248	0.8262
STAGE1_STRIDE_TIME_LEFT	STAGE3_TIME	0.1759	0.1162
STAGE1_STRIDE_TIME_LEFT	STAGE3_SPEED	-0.3055	**0.0056**
STAGE1_STRIDE_TIME_LEFT	STAGE3_ASYMMETRY	0.2518	**0.0233**
STAGE1_STRIDE_TIME_LEFT	STAGE3_STRIDE_TIME_LEFT	0.76	**0.0000**
STAGE1_STRIDE_TIME_LEFT	STAGE3_STRIDE_TIME_RIGHT	0.7877	**0.0000**
STAGE1_STRIDE_TIME_RIGHT	STAGE2_RealSteps	-0.0954	0.3967
STAGE1_STRIDE_TIME_RIGHT	STAGE2_TIME	0.1721	0.1245
STAGE1_STRIDE_TIME_RIGHT	STAGE2_SPEED	-0.4914	**0.0000**
STAGE1_STRIDE_TIME_RIGHT	STAGE2_ASYMMETRY	0.0374	0.7403
STAGE1_STRIDE_TIME_RIGHT	STAGE2_STRIDE_TIME_LEFT	0.5279	**0.0000**
STAGE1_STRIDE_TIME_RIGHT	STAGE2_STRIDE_TIME_RIGHT	0.6429	**0.0000**
STAGE1_STRIDE_TIME_RIGHT	STAGE3_RealSteps	-0.001	0.9926
STAGE1_STRIDE_TIME_RIGHT	STAGE3_TIME	0.2141	0.0550
STAGE1_STRIDE_TIME_RIGHT	STAGE3_SPEED	-0.4995	**0.0000**
STAGE1_STRIDE_TIME_RIGHT	STAGE3_ASYMMETRY	0.0553	0.6236
STAGE1_STRIDE_TIME_RIGHT	STAGE3_STRIDE_TIME_LEFT	0.8588	**0.0000**
STAGE1_STRIDE_TIME_RIGHT	STAGE3_STRIDE_TIME_RIGHT	0.6855	**0.0000**
STAGE2_RealSteps	STAGE2_TIME	0.9372	**0.0000**

STAGE2_RealSteps	STAGE2_SPEED	-0.3842	**0.0004**
STAGE2_RealSteps	STAGE2_ASYMMETRY	-0.4109	**0.0001**
STAGE2_RealSteps	STAGE2_STRIDE_TIME_LEFT	-0.2328	**0.0365**
STAGE2_RealSteps	STAGE2_STRIDE_TIME_RIGHT	-0.2782	**0.0119**
STAGE2_RealSteps	STAGE3_RealSteps	0.2078	0.0627
STAGE2_RealSteps	STAGE3_TIME	0.1571	0.1613
STAGE2_RealSteps	STAGE3_SPEED	-0.4242	**0.0001**
STAGE2_RealSteps	STAGE3_ASYMMETRY	-0.4133	**0.0001**
STAGE2_RealSteps	STAGE3_STRIDE_TIME_LEFT	-0.1667	0.1369
STAGE2_RealSteps	STAGE3_STRIDE_TIME_RIGHT	-0.4096	**0.0001**
STAGE2_TIME	STAGE2_SPEED	-0.5014	**0.0000**
STAGE2_TIME	STAGE2_ASYMMETRY	-0.4689	**0.0000**
STAGE2_TIME	STAGE2_STRIDE_TIME_LEFT	-0.0555	0.6228
STAGE2_TIME	STAGE2_STRIDE_TIME_RIGHT	-0.0732	0.5158
STAGE2_TIME	STAGE3_RealSteps	0.1939	0.0828
STAGE2_TIME	STAGE3_TIME	0.2107	0.0590
STAGE2_TIME	STAGE3_SPEED	-0.5346	**0.0000**
STAGE2_TIME	STAGE3_ASYMMETRY	-0.3882	**0.0003**
STAGE2_TIME	STAGE3_STRIDE_TIME_LEFT	0.0946	0.4007
STAGE2_TIME	STAGE3_STRIDE_TIME_RIGHT	-0.1933	0.0839
STAGE2_SPEED	STAGE2_ASYMMETRY	-0.0597	0.5964
STAGE2_SPEED	STAGE2_STRIDE_TIME_LEFT	-0.3165	**0.0040**
STAGE2_SPEED	STAGE2_STRIDE_TIME_RIGHT	-0.1269	0.2590
STAGE2_SPEED	STAGE3_RealSteps	-0.5246	**0.0000**
STAGE2_SPEED	STAGE3_TIME	-0.5995	**0.0000**
STAGE2_SPEED	STAGE3_SPEED	0.9157	**0.0000**
STAGE2_SPEED	STAGE3_ASYMMETRY	0.0554	0.6231
STAGE2_SPEED	STAGE3_STRIDE_TIME_LEFT	-0.359	**0.0010**
STAGE2_SPEED	STAGE3_STRIDE_TIME_RIGHT	-0.2189	**0.0497**
STAGE2_ASYMMETRY	STAGE2_STRIDE_TIME_LEFT	0.192	0.0859
STAGE2_ASYMMETRY	STAGE2_STRIDE_TIME_RIGHT	0.1056	0.3480
STAGE2_ASYMMETRY	STAGE3_RealSteps	0.3848	**0.0004**
STAGE2_ASYMMETRY	STAGE3_TIME	0.3791	**0.0005**
STAGE2_ASYMMETRY	STAGE3_SPEED	-0.0972	0.3879
STAGE2_ASYMMETRY	STAGE3_ASYMMETRY	0.2627	**0.0178**
STAGE2_ASYMMETRY	STAGE3_STRIDE_TIME_LEFT	0.1057	0.3478
STAGE2_ASYMMETRY	STAGE3_STRIDE_TIME_RIGHT	0.1598	0.1542
STAGE2_STRIDE_TIME_LEFT	STAGE2_STRIDE_TIME_RIGHT	-0.04	0.7227
STAGE2_STRIDE_TIME_LEFT	STAGE3_RealSteps	0.0654	0.5621
STAGE2_STRIDE_TIME_LEFT	STAGE3_TIME	0.2157	0.0531

STAGE2_STRIDE_TIME_LEFT	STAGE3_SPEED	-0.3174	**0.0039**
STAGE2_STRIDE_TIME_LEFT	STAGE3_ASYMMETRY	-0.0816	0.4692
STAGE2_STRIDE_TIME_LEFT	STAGE3_STRIDE_TIME_LEFT	0.5198	**0.0000**
STAGE2_STRIDE_TIME_LEFT	STAGE3_STRIDE_TIME_RIGHT	0.3564	**0.0011**
STAGE2_STRIDE_TIME_RIGHT	STAGE3_RealSteps	-0.1265	0.2603
STAGE2_STRIDE_TIME_RIGHT	STAGE3_TIME	0.0204	0.8568
STAGE2_STRIDE_TIME_RIGHT	STAGE3_SPEED	-0.1419	0.2063
STAGE2_STRIDE_TIME_RIGHT	STAGE3_ASYMMETRY	0.149	0.1844
STAGE2_STRIDE_TIME_RIGHT	STAGE3_STRIDE_TIME_LEFT	0.6279	**0.0000**
STAGE2_STRIDE_TIME_RIGHT	STAGE3_STRIDE_TIME_RIGHT	0.5836	**0.0000**
STAGE3_RealSteps	STAGE3_TIME	0.9688	**0.0000**
STAGE3_RealSteps	STAGE3_SPEED	-0.5443	**0.0000**
STAGE3_RealSteps	STAGE3_ASYMMETRY	0.1738	0.1208
STAGE3_RealSteps	STAGE3_STRIDE_TIME_LEFT	-0.015	0.8939
STAGE3_RealSteps	STAGE3_STRIDE_TIME_RIGHT	0.0513	0.6491
STAGE3_TIME	STAGE3_SPEED	-0.6304	**0.0000**
STAGE3_TIME	STAGE3_ASYMMETRY	0.1681	0.1336
STAGE3_TIME	STAGE3_STRIDE_TIME_LEFT	0.1924	0.0853
STAGE3_TIME	STAGE3_STRIDE_TIME_RIGHT	0.2226	**0.0458**
STAGE3_SPEED	STAGE3_ASYMMETRY	0.094	0.4039
STAGE3_SPEED	STAGE3_STRIDE_TIME_LEFT	-0.3881	**0.0003**
STAGE3_SPEED	STAGE3_STRIDE_TIME_RIGHT	-0.22	**0.0485**
STAGE3_ASYMMETRY	STAGE3_STRIDE_TIME_LEFT	0.1259	0.2626
STAGE3_ASYMMETRY	STAGE3_STRIDE_TIME_RIGHT	0.6187	**0.0000**
STAGE3_STRIDE_TIME_LEFT	STAGE3_STRIDE_TIME_RIGHT	0.6055	**0.0000**
